# Efficient differentiation of human pluripotent stem cells into skeletal muscle cells by combining RNA-based MYOD1-expression and POU5F1-silencing

**DOI:** 10.1038/s41598-017-19114-y

**Published:** 2018-01-19

**Authors:** Tomohiko Akiyama, Saeko Sato, Nana Chikazawa-Nohtomi, Atsumi Soma, Hiromi Kimura, Shunichi Wakabayashi, Shigeru B. H. Ko, Minoru S. H. Ko

**Affiliations:** Department of Systems Medicine, Keio University School of Medicine, Tokyo, 160 Japan

## Abstract

Direct generation of skeletal muscle cells from human pluripotent stem cells (hPSCs) would be beneficial for drug testing, drug discovery, and disease modelling *in vitro*. Here we show a rapid and robust method to induce myogenic differentiation of hPSCs by introducing mRNA encoding MYOD1 together with siRNA-mediated knockdown of POU5F1 (also known as OCT4 or OCT3/4). This integration-free approach generates functional skeletal myotubes with sarcomere-like structure and a fusion capacity in several days. The POU5F1 silencing facilitates MYOD1 recruitment to the target promoters, which results in the significant activation of myogenic genes in hPSCs. Furthermore, deep sequencing transcriptome analyses demonstrated that POU5F1-knockdown upregulates the genes associated with IGF- and FGF-signaling and extracellular matrix that may also support myogenic differentiation. This rapid and direct differentiation method may have potential applications in regenerative medicine and disease therapeutics for muscle disorders such as muscular dystrophy.

## Introduction

Human pluripotent stem cells (hPSCs) such as human embryonic stem cells (hESCs) and induced pluripotent stem cells (hiPSCs) have the potential to differentiate into essentially all the cell types in our body including skeletal muscle cells. hPSC-derived skeletal muscle cells would be an unlimited cell source for potential clinical and research applications such as disease modeling, platform for drug screening, and cell transplantation. Furthermore, there has been hopes of curing genetic disorders such as muscular dystrophies through genetically engineering patient-derived hiPSCs. To this end, it is crucial to develop rapid, efficient, and reliable methods to differentiate hPSCs into skeletal myogenic cells.

For directing myogenic differentiation, previous studies have developed *in vitro* culture conditions of hPSCs using media supplemented with suitable growth factors and cytokines to follow the steps of embryonic development^1,2^. Although those protocols mimic the process of developmental stages, in most cases, they require long-term, complicated steps; yet, the efficiency of differentiation is rather low. To overcome these limitations, recent studies have utilized forced expression of the myogenic master regulator MYOD1 into hPSC-derived mesodermal cells, which can enhance myogenic differentiation. In fact, MYOD1 is the first transcription factor that was identified by its ability to reprogram fibroblast cells into muscle cells^3^. MYOD1 was also demonstrated to induce the conversion of hPSC-derived mesenchymal cells into engraftable myoblast-like cells^4^. Moreover, efficient myogenic differentiation of hiPSC-derived mesoangioblast-like progenitors was accomplished by the overexpression of MYOD1^5^.

In the above studies, MYOD1 was not directly introduced in hPSCs but rather in the mesodermal derivatives, which takes time and needs several differentiation steps to generate from hPSCs. Thus, it has been thought that the direct generation of myogenic cells from hPSCs by using MYOD1 overexpression would be a simple and robust differentiation method. However, MYOD1-directed conversion is much more difficult in hPSCs than in differentiated cells^4,6,7^. Indeed, MYOD1 overexpression in hESCs fails to generate myogenic conversion, whereas comparable levels of MYOD1 expression efficiently induce myogenic differentiation from fibroblast cells^6^. When the combination of a *piggyBac* transposon and drug-inducible expression system induces the high expression of MYOD1, direct myogenic conversion of hiPSCs can be successfully achieved^8^, suggesting that stable and robust expression of MYOD1 proteins is required to activate skeletal myogenesis in hPSCs. Furthermore, recent studies have shown that additional expression of epigenetic modifying factors such as JMJD3 and BAF60C is required to initiate the myogenic program in hPSCs^6,9^. These results suggest that hPSCs are essentially resistant to MYOD1-mediated myogenic differentiation. The pluripotency-gene regulatory network may be involved in the inhibition of direct myogenic differentiation.

Another problem is that most studies described above have employed viral and transposon vectors for overexpression of MYOD1. Although these systems can effectively induce the expression of exogenous genes in hPSCs, they have considerable limitations in therapeutic applications: for example, possible insertional mutagenesis due to random integration into the host genome. We have recently reported that introduction of synthetic mRNA (synRNA) encoding lineage-defining transcription factors can differentiate mouse ESCs into various cell lineages such as neurons, myocytes, hepatocytes, and blood cells^10^. Furthermore, we have generated functional neurons from hPSCs in a week by using an synRNA cocktail of five neuronal transcription factors^11^. This technique eliminates the risk of genomic DNA integration and insertional mutagenesis, and is thus considered suitable for therapeutic applications. Furthermore, the advantages of using synRNA are that it is immediately translated at high expression levels upon entry into cells and that stable expression can be controlled by multiple transfection. It has been demonstrated that sequential transfection of synRNA encoding MYOD1 (synMYOD1) efficiently converts hiPSCs-derived fibroblasts into myogenic cells^12^. However, myogenic differentiation hardly occurs when synMYOD1 is introduced in undifferentiated hPSCs^9^, which corresponds to the results using DNA-based methods.

In this study, we have established a robust RNA-based method to generate skeletal muscle cells directly from undifferentiated hPSCs. First, we found that the expression of a pluripotency master regulator POU5F1 (also known as OCT4 or OCT3/4), but not NANOG, is sustained during the MYOD1-mediated differentiation of hPSCs. We thus silenced the POU5F1 expression by using a small interfering RNA (siRNA) to facilitate the myogenic differentiation induced with synMYOD1. This method has efficiently accomplished the direct differentiation of hPSCs into functional myogenic cells. We also conducted deep sequencing transcriptome analyses to reveal the influence of POU5F1 knockdown on myogenic differentiation.

## Results

### Pluripotent marker POU5F1 stably remains in MYOD1-overexpressing ES cells

**Figure 1:**
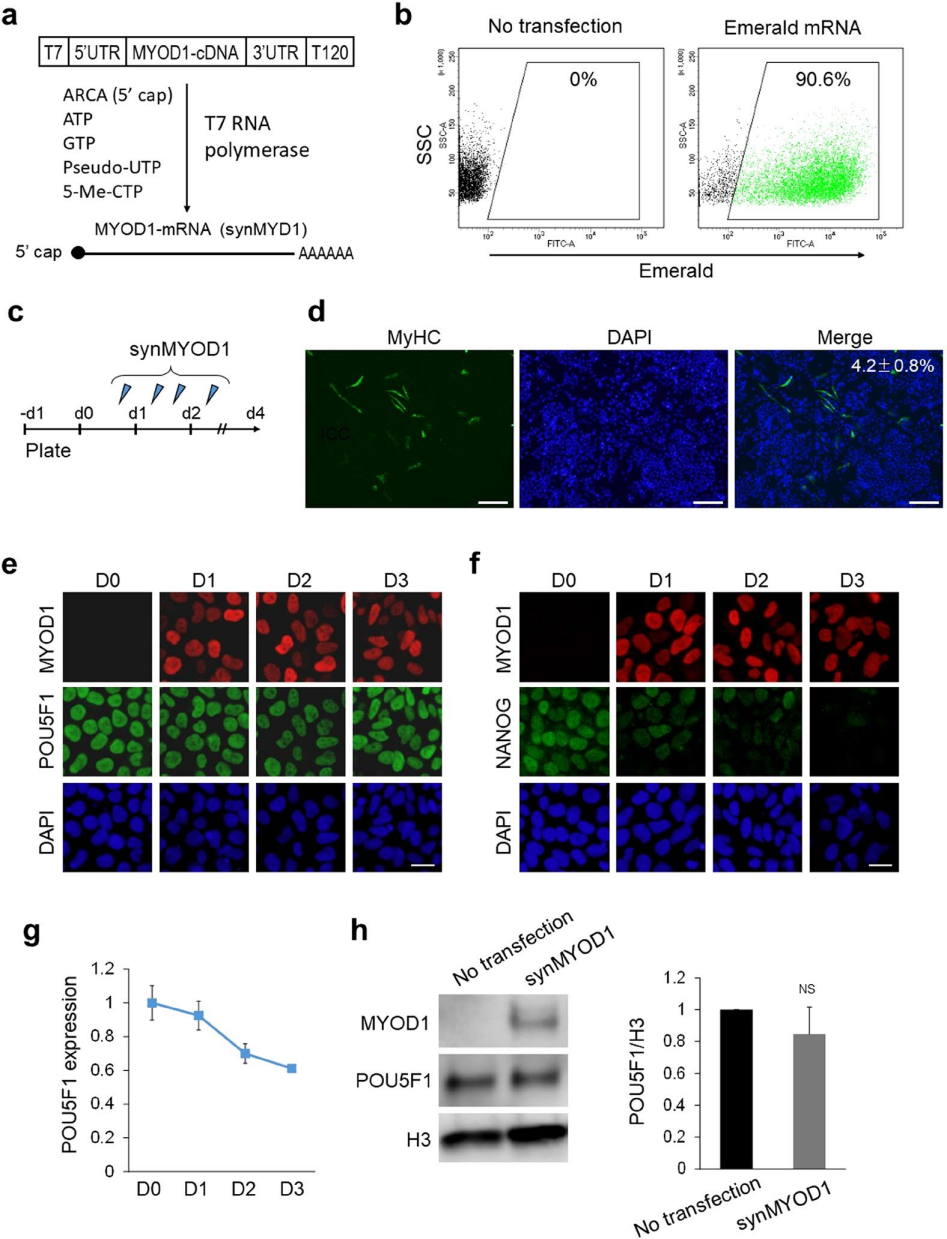
POU5F1 expression is stably sustained in MYOD1-mRNA (synMYOD1)-treated hESCs. (**a**) synMYOD1 was synthesized *in vitro* with T7 RNA polymerase. The template cDNA was flanked by 5′UTR and 3′UTR of alpha-globin with an oligo(T)_120_ for adding a polyA tail. ARCA (5′cap analog), pseudo-UTP, and 5-methyl-CTP were incorporated to increase mRNA stability and translation efficiency. (**b**) The percentage of mRNA transfection in hESCs was tested using synthetic mRNA encoding Emerald GFP by FACS analysis. (**c**) Schematic diagram of the transfection protocol. hESCs were transfected with synMYOD1 once on day 0, twice on day 1, and once on day 2. (**d**) Immunostaining analysis for MyHC in the synMYOD1-transfected cells. Nuclei were stained with DAPI. The percentage of MyHC-stained cells is shown (mean ± SEM from four independent biological replicates). Scale bar: 200 μm. (**e**) Immunostaining analysis for POU5F1 in the synMYOD1-transfected cells at day 0 to day 3 post transfection. MYOD1 was detected by a MYOD1 specific antibody. Nuclei were stained with DAPI. Scale bar: 10 μm. (**f**) Immunostaining analysis for NANOG in the synMYOD1-transfected cells at day 0 to day 3 post transfection. MYOD1 was detected by the specific antibody. Nuclei were stained with DAPI. Scale bar: 10 μm. (**g**) qRT-PCR analysis for POU5F1 expression day 0 to day 3 after transfection (mean ± SEM from two independent biological replicates). (**h**) Immunoblotting analysis for POU5F1 in the synMYOD1-transfected cells at day 3 post transfection. MYOD1 was detected by the specific antibody. The H3 antibody was used as a loading control. The relative intensities of POU5F1 signals normalized by H3 were compared between no transfection and synMYOD1 transfection (mean ± SEM from three independent biological replicates). NS: not significant. Uncropped images of the blots for Fig. 1h are shown in Supplementary Figure 7.

In this study, we generated synthetic RNA encoding MYOD1 *in vitro* (synMYOD1) as reported previously^12^ (Fig. 1a) and transfected them into hPSCs. We achieved ~90% transfection efficiency in hESCs (cell line, SEES3^13^). (Fig. 1b) and comparable transfection efficiency in hiPSCs (cell line, 409B2^14^) (Supplementary Fig. S1). As the protein expression from synRNAs is transient and reaches its peak at 8~18 h after introduction of synRNAs^11,12^, four RNA transfections were performed within two days to maintain the translated protein levels (Fig. 1c). Four days after the first transfection, the myogenic differentiation was assessed by immunostaining analysis of myosin heavy chain (MyHC) – a marker for mature skeletal muscles. As corresponding to previous studies, the efficiency of myogenic conversion was significantly low. Only ~5% of the cells were MyHC-positive (Fig. 1d), whereas Brachyury T (a marker for mesoderm stage) was expressed in most of the MyHC-negative cells (Supplementary Fig S2), suggesting that the majority of synMYOD1-treated cells only reached the mesoderm stage by day 4. It has been known that MYOD1-mediated myogenic conversion efficiently occurs with various differentiated cell types as well as fibroblasts^3,15–17^. Therefore, we hypothesized that the pluripotency-specific factors such as POU5F1 and NANOG inhibit the MYOD1-mediated conversion of hPSCs because these genes are essential for maintenance of the undifferentiated state^18^. To test this hypothesis, we examined the expression states of POU5F1 and NANOG in synMYOD1-transfected hESCs. Immunostaining analysis revealed that the expression of POU5F1 was sustained in MYOD1-expressing cells even at three days post synMYOD1 transfection (Fig. 1e). In contrast, however, the expression of NANOG was decreased the next day after mRNA transfection and it became negligible three days after the transfection (Fig. 1f). The other pluripotency factors - SOX2 and MYC, also showed rapid downregulation in MYOD1-expressing cells (Supplementary Fig. S3). We confirmed, quantitatively by RT-qPCR and immunoblotting analysis, that the expression of POU5F1 decreased gradually, but remained high even by day 3 (Fig. 1g,h). These results suggest that the remaining expression of POU5F1 may inhibit MYOD1-induced myogenic differentiation of hPSCs.

### Knockdown of POU5F1 activates synMYOD1-induced myogenic program

**Figure 2:**
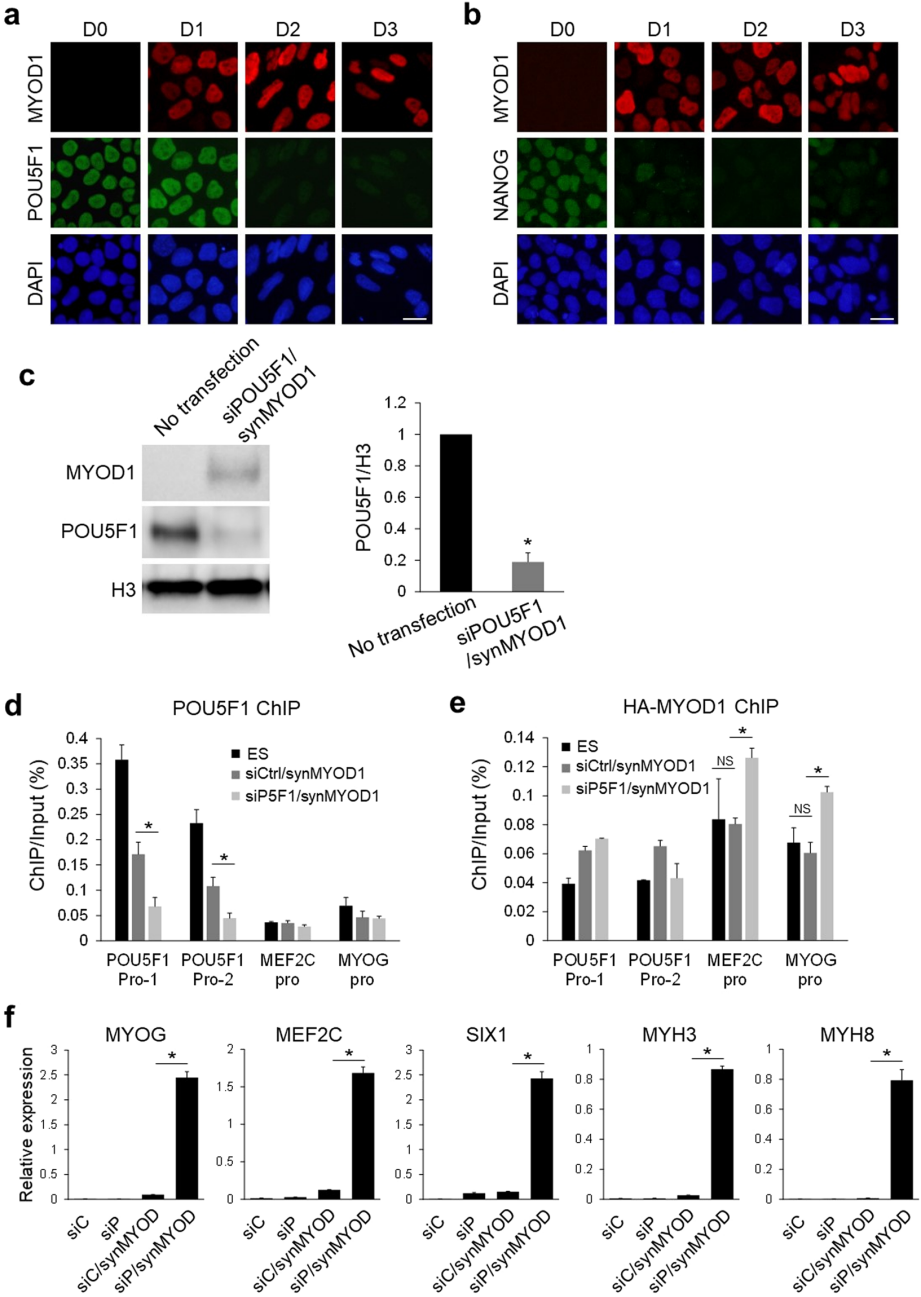
POU5F1 knockdown facilitates synMYOD1-induced myogenic gene activation. (**a**) Immunostaining analysis for POU5F1 in the siPOU5F1 and synMYOD1 (siPOU5F1/synMYOD1)-transfected cells at day 0 to day 3 post transfection. MYOD1 was detected by the specific antibody. Nuclei were stained with DAPI. Scale bar: 10 μm. (**b**) Immunostaining analysis for NANOG in the siPOU5F1/synMYOD1-transfected cells at day 0 to day 3 post transfection. MYOD1 was detected by the specific antibody. Nuclei were stained with DAPI. Scale bar: 10 μm. (**c**) Immunoblotting analysis for POU5F1 in the siPOU5F1/synMYOD1-transfected cells at day 3 post transfection. MYOD1 was detected by the specific antibody. The H3 antibody was used as a loading control. The relative intensities of POU5F1 signals normalized by H3 were compared between no transfection and siPOU5F1/synMYOD1 transfection (mean ± SEM from three independent biological replicates). *P < 0.01, t-test. (**d**) ChIP analysis showing POU5F1 enrichment at the promoter regions (Pro-1 and Pro-2) of POU5F1 in hESCs, siControl/synMYOD1-treated cells, and siPOU5F1/synMYOD1-treated cells. The promoter regions of MEF2C and MYOG were used as negative controls. The error bars indicate the SEM from three independent biological replicates. *P < 0.05, t-test. (**e**) ChIP analysis showing MYOD1 enrichment at the promoter regions of MEF2C and MYOG in hESCs, siControl/synMYOD1-treated cells, and siPOU5F1/synMYOD1-treated cells. mRNA encoding HA-tagged MYOD1 and anti-HA antibody was used in this experiment as the anti-MYOD1 antibody suitable for ChIP was not available. The promoter regions of POU5F1 were used as negative controls. The error bars indicate the SEM from two independent biological replicates. *P < 0.05, t-test. NS: not significant. (**f**) qPT-PCR analysis for the expression of myogenic markers in the siControl (siC), siPOU5F1 (siP), siC/synMYOD1, and siP/synMYOD1-treated cells. The expression levels were normalized to *GAPDH*. The error bars indicate the SEM from two independent biological replicates. *P < 0.01, t-test. Uncropped images of the blots for Fig. 2c are shown in Supplementary Figure 7.

To address the question of whether persisting POU5F1 prevents the myogenic conversion of hPSCs, we tried to downregulate POU5F1 at the same time as MYOD1 overexpression. To follow the RNA-based method, we used a small interfering RNA against POU5F1 (siPOU5F1) to repress the expression of POU5F1 in this experiment. Using the same product of siPOU5F1 as previously used^19^, we could downregulate POU5F1 in hPSCs to undetectable levels, which was confirmed by immunoblotting and immunostaining (Supplementary Fig. S4). This effect of POU5F1 knockdown was sustained for several days with one transfection. When hESCs were transfected with siPOU5F1 together with synMYOD1, POU5F1 expression level was rapidly reduced at day 2 post transfection (Fig. 2a). Moreover, the decrease in NANOG expression was observed immediately after transfection in a similar manner to the condition using synMYOD1 alone (Fig. 2b). The significant reduction of POU5F1 was quantitatively confirmed by immunoblotting analysis (Fig. 2c). The chromatin immunoprecipitation (ChIP) experiment also revealed that transfection of siPOU5F1/synMYOD1 results in the dissociation of POU5F1 from its own promoter regions (Fig. 2d), suggesting that POU5F1 transcription itself becomes silenced. Importantly, the ChIP analysis also revealed that exogenous MYOD1 protein was bound to the downstream gene promoters (MEF2C and MYOG) only when hESCs were treated with both siPOU5F1 and synMYOD1 (Fig. 2e). These results suggest that POU5F1 knockdown facilitates the binding of exogenous MYOD1 to the regulatory regions to activate the myogenic differentiation program. To support this data, we examined the expression changes of myogenic related genes such as MYOG, MEF2C, SIX1, MYH3, and MYH8 after hESCs were treated with siPOU5F1 and synMYOD1. Real-time PCR analyses revealed that these myogenic markers were significantly upregulated by siPOU5F1 and synMYOD1 treatment (Fig. 2f). However, the synMYOD1 alone or siPOU5F1 alone could not induce significant expression of those myogenic markers. These results suggest that the downregulation of POU5F1 is required for MYOD1-mediated myogenic conversion of hPSCs.

### Generation of skeletal muscle cells from ESCs and iPSCs using siPOU5F1 and synMYOD1

**Figure 3:**
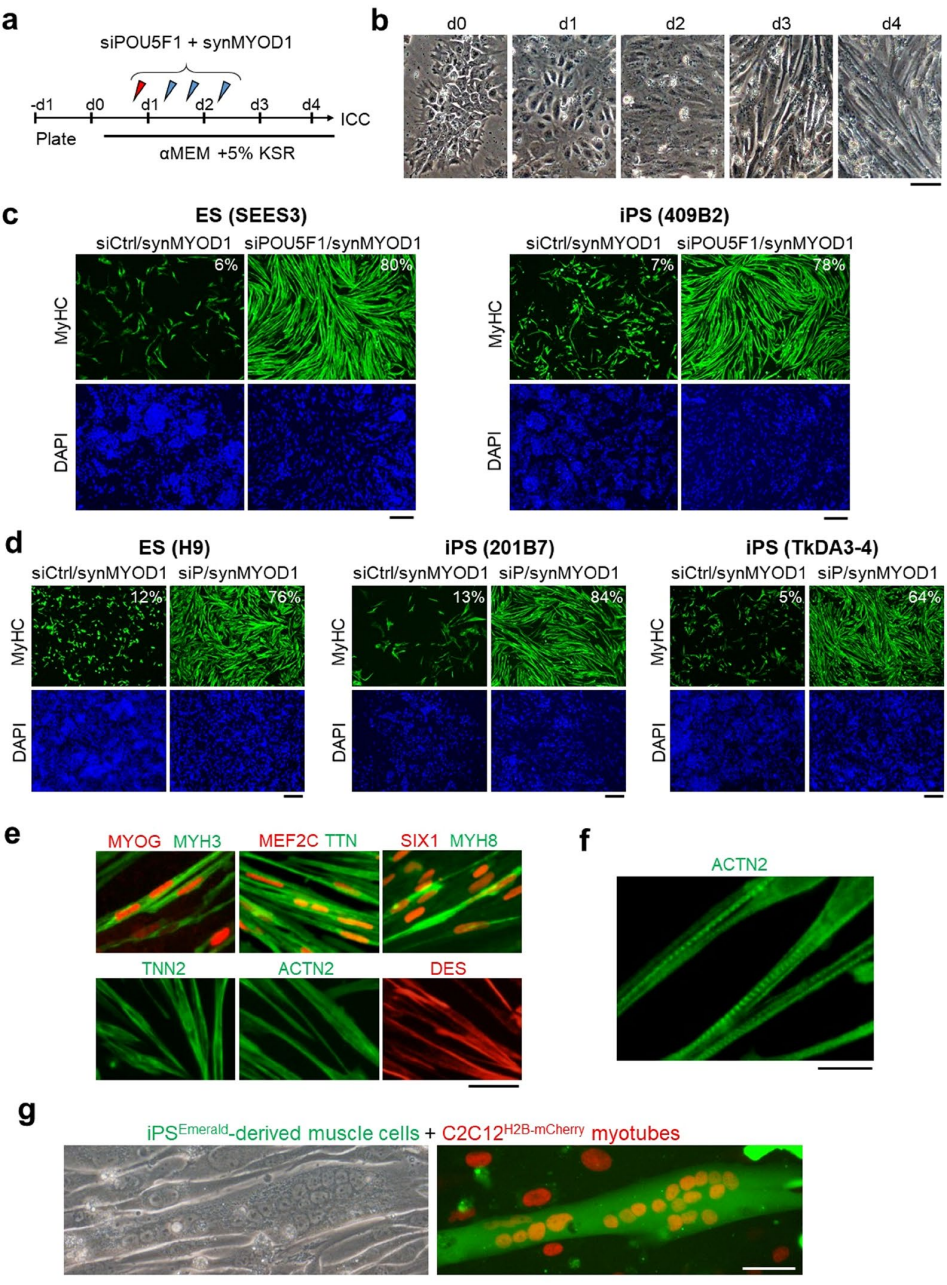
siPOU5F1/synMYOD1 treatment induces efficient myogenic conversion of hPSCs. (**a**) Schematic of the myogenic differentiation protocol. hPSCs were transfected with siPOU5F1 together with synMYOD1 at the indicated time points. The mixture of siPOU5F1/synMYOD1 was transfected on day 0. synMYOD1 was transfected on days 1 and 2. The cells were cultured in αMEM + 5% KSR. (**b**) Morphological changes in the transfected cells. Scale bar, 50 μm. (**c**) Immunostaining analysis for MyHC in the hPSCs (SEES3 ESCs and 409B2 iPSCs) after the treatment with siControl/synMYOD1 or siPOU5F1/synMYOD1. Nuclei were stained with DAPI. The representative images are shown. The average percentages of MyHC-stained cells are obtained from three independent biological replicates. Scale bar: 200 μm. (**d**) Immunostaining analysis for MyHC in the hPSCs (H9 ESCs, 201B7 iPSCs, and TkDA3-4 iPSCs) after treatment with siControl/synMYOD1 or siPOU5F1/synMYOD1. Nuclei were stained with DAPI. The representative images are shown. The average percentages of MyHC-stained cells are obtained from three independent biological replicates. Scale bar: 200 μm. (**e**) Immunostaining analysis for MYOG, MEF2C, SIX1, MYH3, MYH8, titin (TTN), troponin T (TNN2), actinin alpha 2 (ACTN2), and desmin (DES) in the siPOU5F1/synMYOD1-treated cells. Scale bar: 50 μm. (**f**) Higher magnification of ACTN2 staining. Scale bar: 20 μm. (**g**) 409B2-iPSCs expressing Emerald GFP were differentiated into myogenic cells and co-cultured with mouse C2C12 myotubes, nuclei of which were labeled with red fluorescence. Next day after co-culturing, cell fusions were detected. Scale bar: 50 μm.

We next examined if the introduction of siPOU5F1 together with synMYOD1 can efficiently convert hESCs and hiPSCs into terminally-differentiated muscle cells. To this end, siPOU5F1 was transfected once and synMYOD1 were transfected four times in hESCs or hiPSCs (cell lines; ES-SEES3 and iPS-409B2) (Fig. 3a). The transfected cells were cultured in a differentiation medium that was used for myogenic conversion previously^8,9^. As a negative control, a scramble siRNA (siControl) was introduced together with synMYOD1. We confirmed that the differentiation of hESCs or hiPSCs did not occur in the control condition. However, when siPOU5F1 was transfected into hESCs or hiPSCs with synMYOD1, the morphology of the most cells changed dramatically into elongated myogenic-like cells one or two days after the final transfection (day 4 or 5 post differentiation) (Fig. 3b, Supplementary Movie S1). At day 5 post differentiation, we fixed the cells and performed immunostaining analysis to detect the expression of MyHC. We found that muscle-like cells showed high expression of MyHC (Fig. 3c) and the percentage of MyHC-positive cells was much higher in siPOU5F1/synMYOD1-treated cells compared with siControl/synMYOD1-treated cells (*P* < 0.01, Fig. 3c). About 80% of the ESCs and iPSCs were differentiated into myogenic cells by the treatment of siPOU5F1/synMYOD1, whereas myogenic conversion occurs in only less than 10% of the ESCs and iPSCs by the treatment of siControl/synMYOD1. Similar results were obtained with three other pluripotent cell lines - H9 ESCs^20^, 201B7 iPSCs^21^ and TkDA3-4 iPSCs^22^, demonstrating the robustness of this method (Fig. 3d). We also found that the induced skeletal muscle-like cells express other myogenic proteins such as MYOG, MEF2C, SIX1, MYH3/8 (embryonic and perinatal myosin heavy chains), titin (TTN), actinin alpha 2 (ACTN2), troponin T (TNNT2) and desmin (DES) (Fig. 3e). By contrast, PAX7, a marker of myoblasts, and MYH2, a marker of adult myotubes, were not detected in these cells (Supplementary Fig. S5), suggesting that hPSCs were converted into fetal or perinatal myotube-like cells. Notably, the localization of ACTN2 showed the periodical patterns in the cytoplasm (Fig. 3f), indicating that the sarcomere structure – the basic contractile unit of muscle tissue seemed to be generated in the induced myogenic cells. Indeed, when those cells were further differentiated in 2% horse serum medium, myofiber-like cells were generated and exhibited spontaneous contractions (Supplementary Movie S2). Furthermore, co-culturing with mouse C2C12 myoblast clearly showed that the induced myogenic cells possess fusion capability (Fig. 3g). Taken together, these results demonstrated the functionality of the myotube cells induced by the RNA-based method using siPOU5F1 and synMYOD1.

### Knockdown of POU5F1 directly regulates early myogenic genes but not the late-stage genes

**Figure 4:**
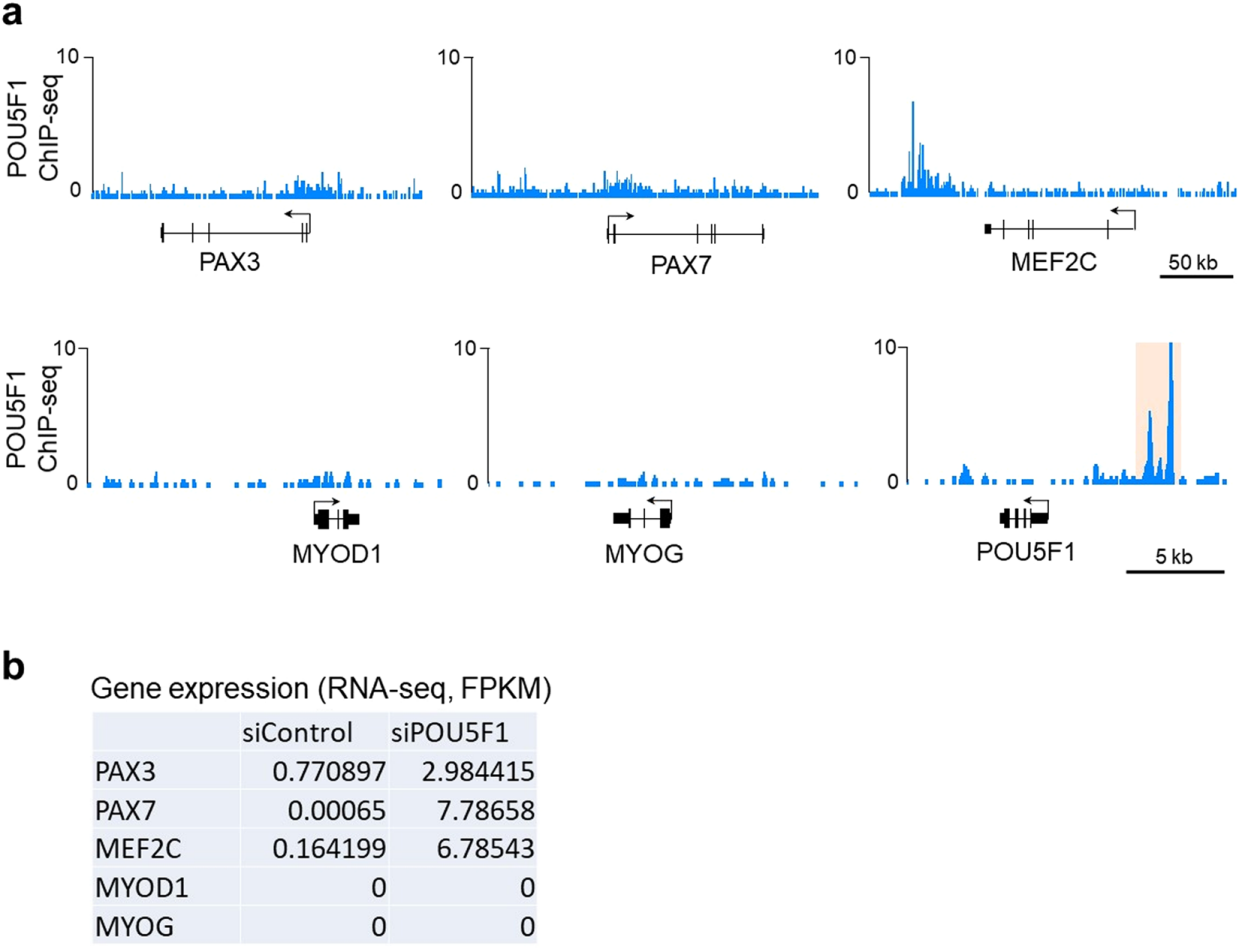
POU5F1 binding profiles around the myogenic gene loci and the effect of POU5F1 knockdown on the expression of the genes. (**a**) ChIP-sequencing tracks of POU5F1 for the loci of myogenic genes: PAX3, PAX7, MEF2C, MYOD1, and MYOG. The POU5F1 locus is shown as a positive control for the POU5F1 binding sites (orange). The data was obtained from a previous study^24^ (**b**) Expression levels of myogenic genes in the siControl- and siPOU5F1-treated cells were analyzed by RNA-sequencing. The FPKM values are shown.

We next tried to explore the direct contribution of POU5F1 knockdown on myogenic gene activation in hPSCs. A previous study reported that ectopic expression of Pou5f1 suppresses the myogenic genes such as Pax7 and Myod1 in mouse myoblasts^23^. The study also demonstrated that Pou5f1 directly binds the Myod1 enhancer to suppress Myod1 expression. These results suggested the possibility that endogenous POU5F1 also suppresses the myogenic genes in hPSCs through binding to their regulatory regions, and that the removal of POU5F1 induces the de-repression of the myogenic genes. These mechanisms may support MYOD1-mediated myogenic differentiation. To investigate the possibility, we first examined the binding profiles of POU5F1 on the hESC genome with the ChIP-sequencing data derived from a previous study^24^. Although the POU5F1 binding signals were slightly detected in the promoters of PAX3 and PAX7 genes, there was little to no POU5F1 enrichment in the promoters or enhancers of MYOD1 and MYOG genes (Fig. 4a). POU5F1 binding was detected in the 3’ regions of MEF2C, which may be a putative enhancer of MEF2C. To examine the expression changes of those genes by POU5F1 knockdown, we performed RNA-sequencing analysis and compared the transcriptome profiles between siPOU5F1-treated cells and untreated cells. We found that siPOU5F1 treatment resulted in the upregulation of PAX3, PAX7 and MEF2C, whereas the expression of MYOD1 and MYOG remained silent after siPOU5F1 treatment (Fig. 4b). These results suggest that POU5F1 directly suppresses the early myogenic genes, but not the late myogenic markers in hPSCs.

### POU5F1-knockdown-activated genes support MYOD1-mediated myogenic differentiation

**Figure 5:**
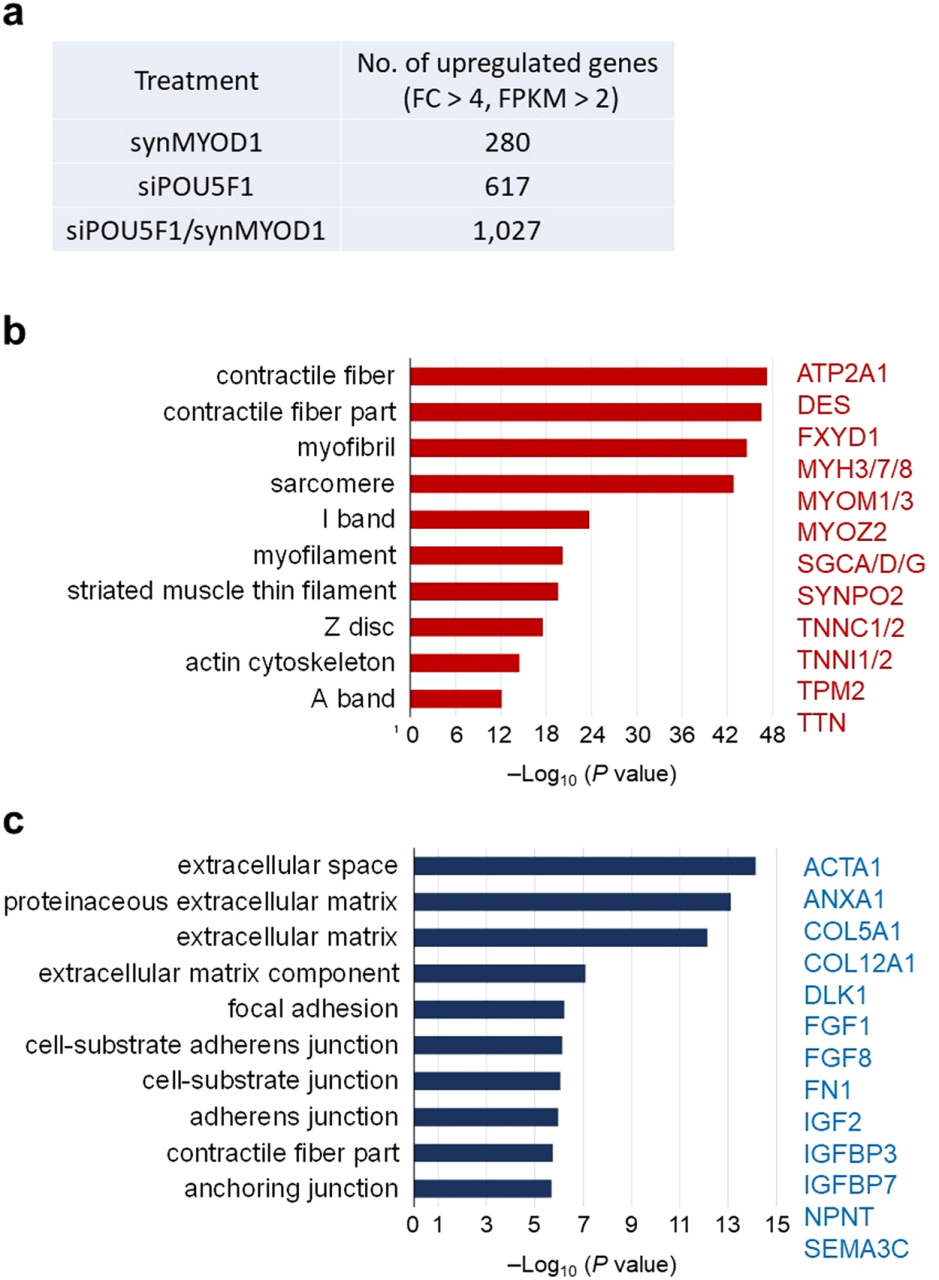
Transcriptome analysis of the synMYOD1-, siPOU5F1-, and siPOU5F1/synMYOD1-treated cells. (**a**) The number of upregulated genes in the synMYOD1-, siPOU5F1-, and siPOU5F1/synMYOD1-treated cells (fold change (FC) > 4 compared with the control ESCs, FPKM values > 2). The number of total genes examined is 20,563. (**b**) Functional annotation analysis of the upregulated genes following siPOU5F1/synMYOD1 transfection. The highly upregulated genes (289 genes, FC > 10 compared with the control ESCs, FPKM > 10) were analyzed to annotate the GO terms related to the cellular component. The *P*-value indicates the significance of the GO term enrichment. Representative genes are shown. (**c**) Functional annotation analysis of the upregulated genes following siPOU5F1 transfection that were also upregulated in the siPOU5F1/synMYOD1 treatment (273 genes). The genes were analyzed to annotate the GO terms related to the cellular component. The *P*-value indicates the significance of the GO term enrichment. Representative genes are shown.

**Figure 6:**
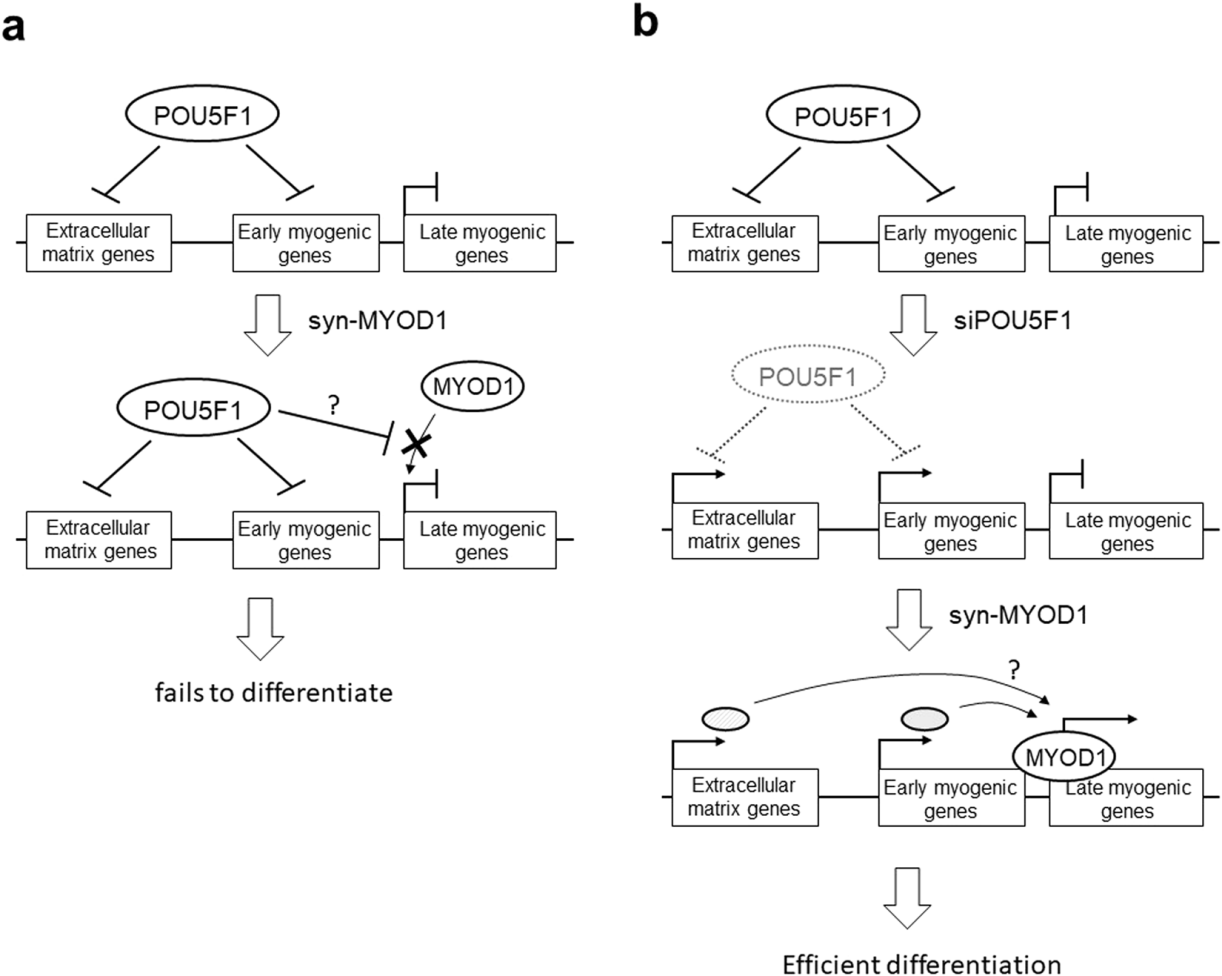
A model of the mechanism for the effective myogenic differentiation of hPSCs with siPOU5F1 and synMYOD1. (**a**) In hPSCs, POU5F1 directly represses the expression of the genes related to extracellular matrix and early myogenic genes such as PAX3 and PAX7. Additionally, when synMYOD1 alone is introduced in hPSCs, POU5F1 inhibits the access of the translated MYOD1 protein to the target late myogenic genes such as MEF2C and MYOG, which results in the failure of myogenic differentiation. (**b**) Knockdown of POU5F1 with siPOU5F1 causes the activation of extracellular matrix genes and early myogenic genes. Consequently, when synMYOD1 is introduced, the translated MYOD1 protein can access to the promoters of the late myogenic genes to activate terminal myogenic gene activation. These mechanisms facilitate the rapid and highly efficient myogenic differentiation in hPSCs.

To further explore the effects of siPOU5F1 on the transcriptome changes that contribute to efficient myogenic conversion, we compared the upregulated genes among siPOU5F1-, synMYOD1-, and siPOU5F1 plus synMYOD1 (siPOU5F1/synMYOD1)-treated cells with RNA-sequencing analysis. siControl-treated or Emerald-mRNA-treated cells were used as the relative control for transcript levels. In the synMYOD1-treated cells, there were 280 upregulated genes (fold change > 4 compared to the control, FPKM > 2) (Fig. 5a) that contain mesodermal genes and myogenic genes such as T, MSX1, RYR1, MYL1/2/4, and DES (Supplementary Table S1). However, the expression levels of those genes were relatively low (FPKM < 10), which may be the reason for low efficiency of myogenic differentiation. We found that siPOU5F1/synMYOD1 treatment upregulated 1,027 genes (fold change > 4 compared to the control ESCs, FPKM > 2) (Fig. 5a) which also includes many myogenic genes (Supplementary Table S2). To extract the genes that actually contribute to myogenic differentiation, we focused on the more highly-upregulated genes (fold change > 10, FPKM > 10). By this criteria, 289 genes were detected in siPOU5F1/synMYOD1-treated cells whereas only 22 genes were detected in synMYOD1 condition. We found that more than 100 genes related to myogenesis were included in the highly-upregulated genes of siPOU5F1/synMYOD1 treatment (Supplemental Table S3). Importantly, these genes contained the terminal differentiation makers associated with contractile fiber and sarcomere structure, such as MYOM1/3, MYH3/7/8, ATP2A1, DES, SGCA/D/G, TNNC1/2, TNNI1/2, and TTN (Fig. 5b). The expression levels of those genes were comparable to those of myotubes differentiated from myoblast cells (cell line, HSMM) (Supplementary Fig. S6). We reasoned that this myogenic gene activation was enhanced by the addition of siPOU5F1 treatment. To reveal the direct influence of siPOU5F1 treatment on myogenic differentiation, we focused on the upregulated genes of siPOU5F1 treatment that were also upregulated in the siPOU5F1/synMYOD1 treatment. Out of 617 total genes, 273 genes were contained in this group (Supplemental Table S4). Significantly, we found that those genes include those associated with IGF- and FGF-signaling and the extracellular matrix, such as IGF2, IGFBP3, FGF1/8, DLK1, ACTA1, FN1, COL5A1, and COL12A1 (Fig. 5c). These genes are known to be important for muscle differentiation or are highly expressed in adult myogenic cells^9,25–29^. Therefore, these results suggest that knockdown of POU5F1 expression contributes to the generation of an intra/extracellular environment that enables MYOD1 and the downstream genes to activate the myogenic program, which may enhance the direct conversion of hPSCs into skeletal muscle cells (see Fig. 6 for the model).

### POU5F1-knockdown is effective for differentiation of other lineages from hPSCs

**Figure 7:**
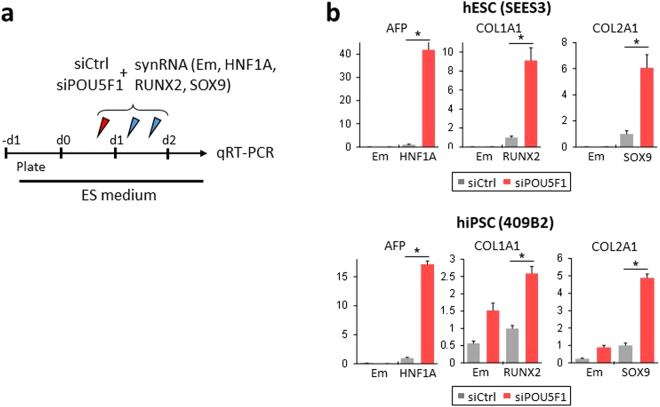
siPOU5F1 facilitates the upregulation of relevant tissue-specific markers mediated by other transcription factor synRNAs. (**a**) Experimental schematic for (**b**). hESC/iPSCs were transfected with siCtrl or siPOU5F1 and synRNAs for HNF1A, RUNX2, and SOX9 at the indicated time points. Emerald synRNA was used as a control. A mixture of siRNA and synRNA was transfected on the first day. On day 2, only synRNA was transfected twice. The cells were cultured in ES maintenance medium and sampled for qRT-PCR analysis on day 2. (**b**) Relative gene expression levels of tissue-specific genes (AFP, COL1A1, and COL2A1) in hESCs (SEES3) and hiPSCs (409B2) transfected with siCtrl or siPOU5F1 and synRNA. The transfected synRNA of transcription factors are indicated on the horizontal axis. Em indicates Emerald synRNA. The expression levels were normalized against that of GAPDH. Error bars indicate SEM (n = 3). *P < 0.05, t-test.

Lastly, to test whether knockdown of POU5F1 could also be applied to differentiation of other lineages from hPSCs, we introduced synRNAs encoding lineage-defining transcription factors - HNF1A, RUNX2, and SOX9 into siPOU5F1-treated hESCs/hiPSCs (Fig. 7a). It has been known that HNF1A, RUNX2, and SOX9 are important in the differentiation of hepatogenesis^30^, osteogenesis^31^, and chondrogenesis^32^, respectively. We analyzed the expression changes in the downstream genes of these transcription factors at two days post transfection. Real-time RT-PCR analyses showed that when the transcription factors were overexpressed in siPOU5F1-treated cells, relevant differentiation markers (AFP for hepatogenesis, COL1A1 for osteogenesis, COL2A1 for chondrogenesis) were highly expressed compared with the siCtrl-treated cells (Fig. 7b). siPOU5F1 alone (synEmerald was introduced) did not change the expression patterns of the tissue-related genes. These results suggest that POU5F1-knockdown does not directly affect the expression of tissue-specific genes, but instead facilitates their activation, which is mediated by lineage-defining transcription factors.

## Discussion

In this study, we have shown a rapid and highly efficient method to generate functional skeletal muscle cells from hPSCs by introducing an mRNA encoding MYOD1 and an siRNA against POU5F1. Our method combines simplicity and robustness: Gene induction is applied by RNA transfection, and genetic manipulation is not required. RNA transfection has merit over DNA transfection for potential therapeutic application, because it has no risk of integration into the host genome. Furthermore, our method does not require the design of complex plasmid constructs or virus packaging, although it requires repeated RNA transfection to maintain the stable protein expression. In addition to this simplicity, our data have demonstrated efficient (>80%) and rapid (4 days) differentiation of hPSCs. Previous methods require multiple steps and take a few weeks or more to generate muscle cells from hPSCs, including the procedure for generating stable cell lines. Thus, our method facilitates rapid assessments of the efficiency of myogenic differentiation of hPSCs. In addition, this method is suitable for pathological analysis and drug screening assays, which require the use of many different hPSC lines that are specifically made for different types of diseases.

Our data showed that POU5F1 expression is stably sustained after introduction of MYOD1-mRNA and inhibits myogenic differentiation in hPSCs. The siRNA-mediated POU5F1 knockdown enabled MYOD1 to activate the expression of terminal myogenic markers and enhanced the myogenic differentiation. Because POU5F1 does not bind the regulatory elements of MYOD1 gene or other myogenic genes and does not directly inhibit the gene expression related to muscle differentiation, POU5F1 knockdown may indirectly support MYOD1-induced differentiation. Previous studies reported that POU5F1 knockdown upregulates the genes associated with early developmental and the patterns of upregulated genes depend on culture conditions and cell lines^33^. Our transcriptome analysis revealed that POU5F1 knockdown activates myogenic precursor regulators PAX3 and PAX7. We also found that POU5F1 knockdown significantly upregulated the genes associated with IGF2- and FGF-signaling and extracellular matrix such as FN1 (fibronectin), SEMA3 (semaphorin) and DLK1 that are necessary for myogenic development and differentiation^25–29,34^. These genes may support the differentiation of hPSCs by establishing a myogenesis regulatory network in cooperation with MYOD1. In addition, the siPOU5F1-upregulated genes may play a role in facilitating the recruitment of MYOD1 to the target promoters of myogenic regulatory genes. Indeed, JMJD3, which removes repressive histone marks from promoters, was activated by siPOU5F1 treatment (Supplementary Table S4), suggesting that knockdown of POU5F1 changes chromatin structure to induce differentiation.

Knockdown of POU5F1 can also be applied to differentiation of other lineages from hPSCs. The removal of POU5F1 leads to the loss of pluripotency and initiation of the early differentiation program, but the differentiation lineage is not defined yet. Therefore, introduction of lineage-defining transcription factors such as MYOD1 should be necessary to induce a specific tissue differentiation program. Indeed, when an mRNA encoding HNF1A, RUNX2, and SOX9, which are known as an important transcription factors for hepatogenesis^30^, osteogenesis^31^, and chondrogenesis^32^, respectively, are introduced in siPOU5F1-treated conditions, the relevant tissue-specific genes are highly induced in hPSCs. These results suggest that siPOU5F1 treatment may facilitate tissue-differentiation of hPSCs directed by lineage-defining transcription factors.

Collectively, our data demonstrated the robustness of the RNA-based method to differentiate hPSCs into skeletal myogenic cells by introducing POU5F1-siRNA together with MYOD1-mRNA. Because the combinatory introduction of siRNA and mRNA is an integration-free and virus-free approach to easily control the gene expression and differentiation of hPSCs, our strategy could be adapted to enhance the utility of hPSCs in disease modeling, drug screening, and cell transplantation therapy for various types of differentiated cells.

## Methods

### hPSC culture

SEES-3 cells were obtained from the Center for Regenerative Medicine, National Research Institute for Child Health and Development, Japan. H9 hESCs were obtained from the WiCell Research Institute, USA. The hiPSC lines (409B2 and 201B7) were obtained from the RIKEN Bioresource Center, Japan, and the TkDA3-4 line was obtained from the Center for iPS Cell Research and Application, Kyoto University, Japan. The hESCs/iPSCs were maintained in feeder-free conditions using StemFit AK-03/AK-02N medium (Ajinomoto) on iMatrix-511 (Nippi)-coated plates. For myogenic differentiation, the hPSCs were cultured in a medium comprising αMEM (Gibco) supplemented with 5% KnockOut Serum Replacement, 1 mM sodium pyruvate, 0.1 mM non-essential amino acids, 2 mM glutamine, 0.1 mM β-mercaptoethanol, and penicillin/streptomycin (100 U/100 µg/ml) on iMatrix-511-coated plates. All experiments were performed in accordance with the Guidelines for Derivation and Utilization of Human Embryonic Stem Cells by the Ministry of Education, Culture, Sports, Science, and Technology, Japan.

### siRNA, modified RNA synthesis, and transfection

siPOU5F1 (Silencer Select ID s10873) and siControl were obtained from Life Technologies. The ORFs for MYOD1, HA-tagged MYOD1 and Emerald GFP were subcloned into a pCRII construct containing the 5’ untranslated region (UTR) and 3’ UTR of mouse alpha-globin, which increases mRNA stability and translation efficiency, to prepare the templates used for synthesizing the mRNAs. The modified mRNAs were synthesized as described previously^12^. RNA transfection was performed with Lipofectamine Messenger Max (Invitrogen), according to the manufacturer’s instructions. B18R interferon inhibitor (eBioscience) was added to the culture medium to increase the viability of the transfected cells. The medium was replaced 3 h after each transfection. The efficiency of transfection was analyzed using a BD FACSAria II.

### Antibodies

The following antibodies were used: POU5F1 (Abcam #ab19857 and Santa Cruz #sc-5279), NANOG (Abcam #ab21624), MyHC (R&D #MAB4470), SOX2 (Millipore #AB5603), MYC (Abcam #ab32072), T (R&D #AF2085), PAX7 (Invitrogen #PA1-117), MYOG (Abcam #ab124800), MEF2C (CST #5030, DSHB #1D4), SIX1 (CST #12891), MYH2 (Sigma #M1570), MYH3 (DSHB #F1.652), MYH8 (DSHB #N3.36), TTN (DSHB #9D10), ACTN2 (Sigma #A7811), DES (Abcam #ab32362), TNNT2 (Santa Cruz #sc-20025), MYOD1 (BD #554130 and Abcam #ab21624), HA (Abcam #ab18181), and H3 (Abcam #ab1791), and β-actin (Cell Signaling #4970S).

### Immunostaining

The cells were fixed in 4% paraformaldehyde (PFA) for 10 minutes at room temperature (RT) and permeabilized in 0.5% Triton X-100 in PBS for 10 minutes. The cells were blocked in PBS and 2% bovine serum albumin (BSA) for 10 minutes and incubated with the primary antibodies in a blocking solution (1:500) for 2-3 h at RT or overnight at 4 °C. After two washes in PBS, the cells were incubated with Alexa 488 and 594-conjugated secondary antibodies (Invitrogen) in a blocking solution (1:500) for 1 h at RT. Nuclei were counterstained with DAPI (Dako) for 5 min at RT. Immunofluorescence was visualized with an inverted fluorescence microscope IX73 (Olympus). Images were obtained using Olympus cellSens imaging software.

### Immunoblotting

The cells were lysed with a sample buffer (50 mM Tris-HCl, pH 6.8, 2% SDS, 6% 2-mercaptoethanol and 500 mg/ml urea). The proteins were separated by SDS-PAGE on a 4-15% polyacrylamide gel (Bio-Rad) and were electrically transferred to polyvinylidene difluoride membranes (Bio-Rad). The membranes were blocked for 1 h in Tris-buffered saline containing 0.1% Tween-20 (TBST) and 5% skimmed milk. The membranes were washed in TBST and then incubated with primary antibodies in TBS/2% BSA (1:1000) overnight at 4 °C. The membranes were washed and then incubated with horseradish peroxidase-conjugated secondary antibodies (GE Healthcare) (1:2000) for 1 h at RT. The membranes were washed in TBST, and immunoreactivity was visualized using an ECL Prime Detection Kit (GE Healthcare) and detected using a Luminescent Image Analyzer (LAS-4000; Fujifilm). Immunoblots were quantified using ImageJ software. The signal intensity levels were normalized to the loading controls, and the average values were calculated from three independent experiments.

### qRT-PCR

Total RNA was isolated with TRIzol reagent (Invitrogen), and cDNAs were generated with random hexamers using the ReverTra Ace kit (Toyobo). Real-time PCR was performed using a SYBR Green PCR system (Takara). The primer sequences used for RT-PCR are listed in Supplementary Table S5.

### ChIP

The cells were cross-linked with formaldehyde in PBS (final concentration is 1%) for 10 min. The reaction was stopped by glycin (final concentration 125 mM). The cells were lysed in Lysis buffer 3 (10 mM Tris-HCl, pH8.0, 100 mM NaCl, 1 mM EDTA, 0.5 mM EGTA, 0.1% Na-deoxycholate, 0.5% N-lauroylsarcosine) containing proteinase inhibitor cocktail. Sonication was conducted with the Handy Sonic UR-20P (Tomy) to generate DNA fragments of approximately 150–450 bp. The sheared chromatin was precipitated with 3 μg of anti-POU5F1 antibodies or anti-HA antibodies using the ChIP-IT Express kit (Active motif), according to the manufacturer’s instructions. The purified DNA was subject to Real-time PCR with the SYBR Green PCR system (Takara). Primer sequences are listed in Supplementary Table S5.

### RNA-sequencing

The cDNA libraries of siControl-treated cells, siPOU5F1-treated cells, and siPOU5F1/synMYOD1-treated cells were prepared from 500 ng of each total RNA sample for massive parallel sequencing using an NEBNext Poly(A) mRNA Magnetic Isolation Module and an Ultra Directional RNA Library Prep Kit for Illumina (NEB). The cDNA library produced ranged from 400 to 1000 bp, including the adaptor sequences. RNA-seq was performed with an Illumina MiSeq for 150 single-ended base pairs. The sequence reads were mapped to the human genome (hg19) using TopHat v2.0.13. The expression values for genes were calculated as fragments per kilobase of exon per million mapped reads (FPKM) using Cufflinks v2.1.1. The FPKM values of the synMYOD1-treated cells and the control (Emerald-mRNA-treated ESCs) were derived from previous data^9^. FPKM value 0 was set to 0.1, to be able to avoid erratic deviations in fold-change values. The functional annotation of the genes was performed using ToppGene Suite^35^.

### Cell fusion assay

C2C12 myoblast cells expressing H2B-mCherry were differentiated in a differentiation medium consisting DMEM-F12 (Gibco) supplemented with 2% horse serum with IGF for 5 days to generate skeletal myotubes. The 409B2-hiPSCs stably expressing Emerald were differentiated into myogenic cells with siPOU5F1/synMYOD1 treatment. The hiPSCs-derived myogenic cells were co-cultured with the C2C12 myotubes in the differentiation medium. The fused cells were identified by green-fluorescence in the cytoplasm and red-fluorescence in the mutinuclei.

## Electronic supplementary material

Supplementary Movie S1

Supplementary Movie S2

Supplementary Information

Supplementary Table S1

Supplementary Table S2

Supplementary Table S3

Supplementary Table S4

Supplementary Table S5
