# Supplementary material for: Efficient differentiation of human pluripotent stem cells into skeletal muscle cells by combining RNA-based MYOD1-expression and POU5F1-silencing
Source: Sci Rep. 2018 Jan 19;8:1189. doi: 10.1038/s41598-017-19114-y (PMC5775307; doi:10.1038/s41598-017-19114-y)
Supplement: Supplementary file 3 — Supplementary Information [file 41598_2017_19114_MOESM3_ESM.pdf]

Efficient differentiation of human pluripotent stem cells into skeletal muscle cells by combining RNA-based MYOD1-expression and POU5F1-silencing

Tomohiko Akiyama, Saeko Sato, Nana Chikazawa-Nohtomi, Atsumi Soma, Hiromi Kimura, Shunichi Wakabayashi, Shigeru B.H. Ko, Minoru S.H. Ko*

Department of Systems Medicine, Keio University School of Medicine, Tokyo 160, Japan

* Correspondence: Minoru S.H. Ko, M.D., Ph.D. (ko.minoru@keio.jp)

Supplementary Figure S1 (Akiyama et al)

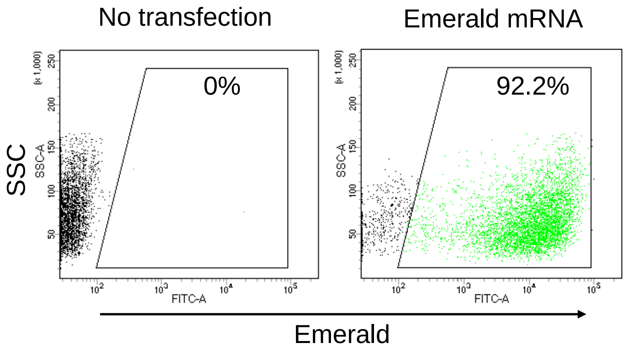

Supplementary Figure S1. Synthetic mRNA transfection in hiPSCs. The percentage of mRNA transfection in 409B7-hiPSCs was tested using synthetic mRNA encoding Emerald GFP by FACS analysis.

Supplementary Figure S2 (Akiyama et al)

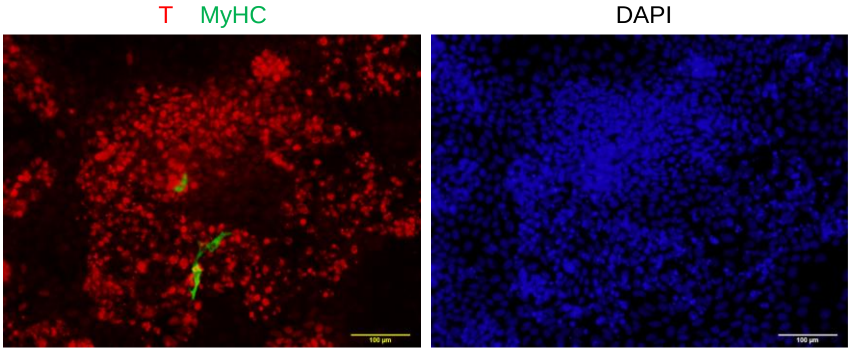

Supplementary Figure S2. Expression of T and MyHC in the synMYOD1-transfected cells. Immunostaining analysis for Brachyury T (a mesoderm marker) and MyHC in the synMYOD1-transfected cells. Nuclei were stained with DAPI.

Supplementary Figure S3 (Akiyama et al)

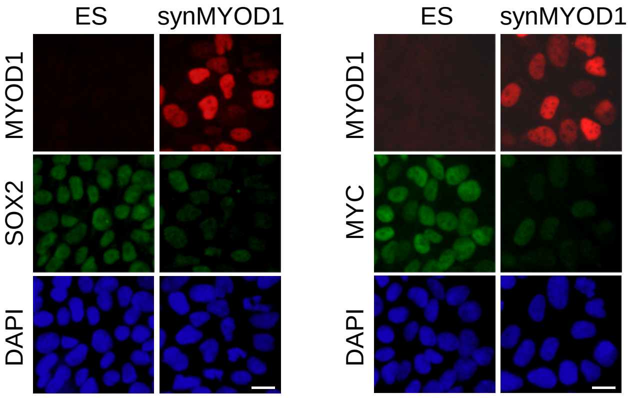

Supplementary Figure S3. Expression of SOX2 and MYC1 in the synMYOD1-transfected cells. Immunostaining analysis for SOX2 and MYC in the synMYOD1-transfected cells at day 1 post transfection. MYOD1 was detected by the specific antibody. Nuclei were stained with DAPI. Scale bar: 10 μm.

Supplementary Figure S4 (Akiyama et al)

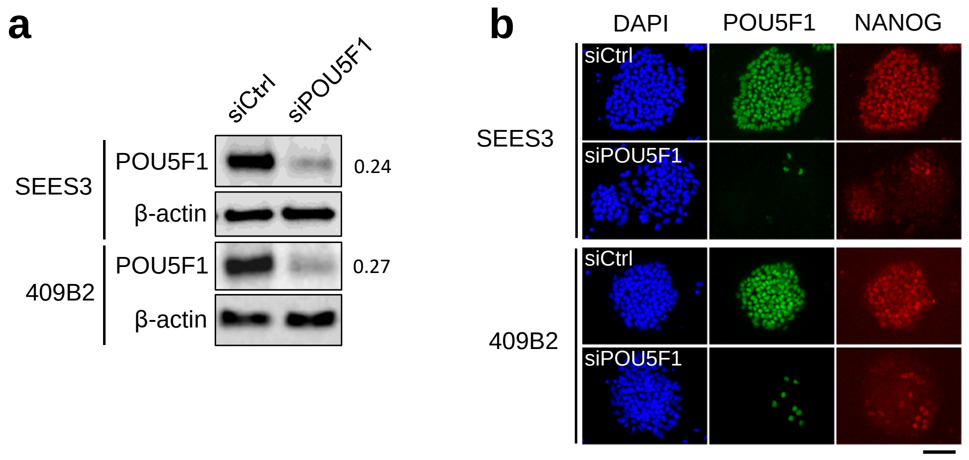

Supplementary Figure S4. POU5F1 knockdown with siPOU5F1 treatment. (**a**) Immunoblotting to detect POU5F1 in siCtrl- and siPOU5F1-treated hESCs (SEES3) and hiPSCs (409B2). The average intensities of siPOU5F1 relative to siCtrl are shown (n = 3). *p < 0.05 by t test. (**b**) Immunostaining to detect POU5F1 and NANOG in siCtrl- and siPOU5F1-treated ESCs (SEES3), and iPSCs (409B2). Scale bar, 100 µm.

Supplementary Figure S5 (Akiyama et al)

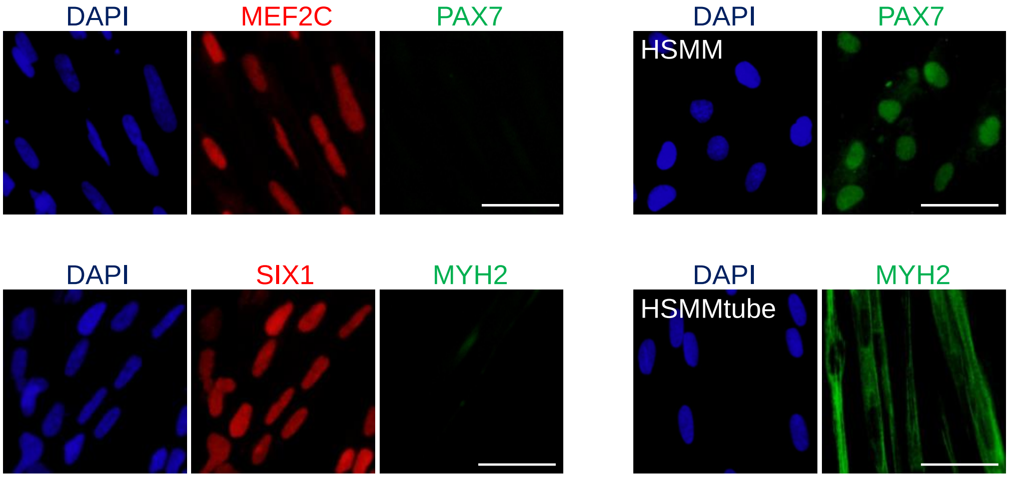

Supplementary Figure S5. Expression of PAX7 and MYH2 in the siPOU5F1/synMYOD1-transfected cells. Immunostaining analysis showing no or faint signals of PAX7 (a myoblast precursor marker) and MYH2 (an adult myotube marker) in the siPOU5F1/synMYOD1-treated cells. The HSMM myoblasts and the differentiated myotubes (HSMMtube) were used as positive controls for PAX7 and MYH2 staining, respectively. Nuclei were stained with DAPI. Scale bar: 50 µm.

Supplementary Figure S6 (Akiyama et al)

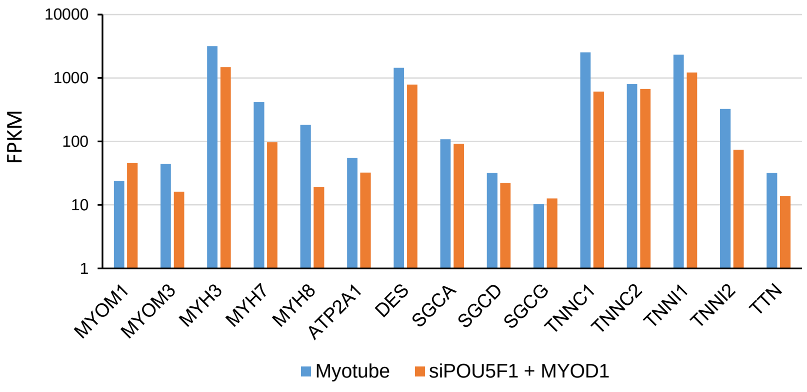

Supplementary Figure S6. Comparison of myogenic gene expression between cultured myotubes and siPOU5F1/synMYOD1-induced muscle. FPKM scores of indicated genes are shown. RNA-seq data of myotubes differentiated from myoblast cells (ref. 9) was used.

Supplementary Figure S7 (Akiyama et al)

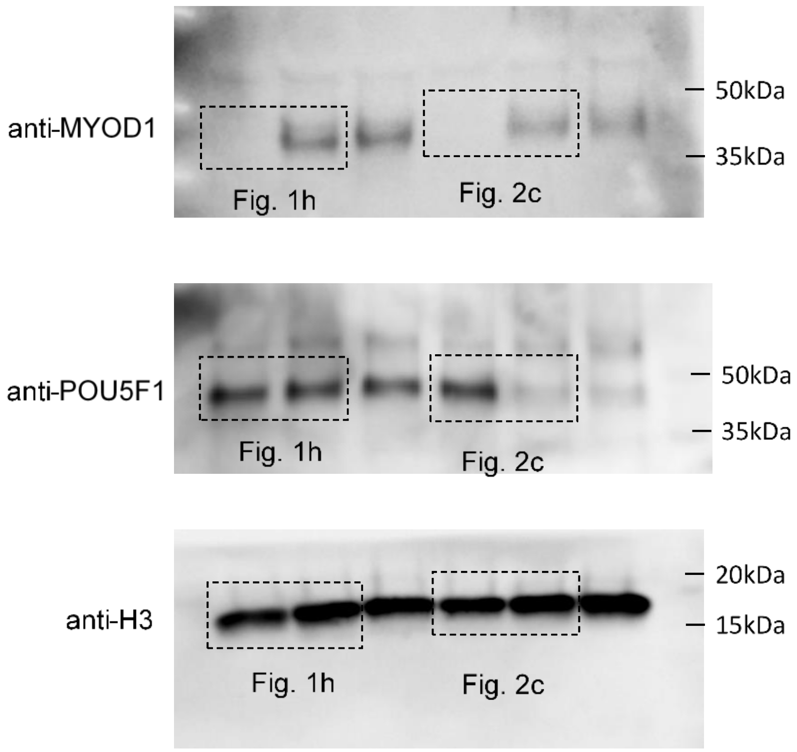

Supplementary Figure S7. Original images of immune blots for indicated figures.
